# Effect of Tenofovir Disoproxil Fumarate and Emtricitabine on nasopharyngeal SARS-CoV-2 viral load burden amongst outpatients with COVID-19: A pilot, randomized, open-label phase 2 trial

**DOI:** 10.1016/j.eclinm.2021.100993

**Published:** 2021-06-27

**Authors:** Jean-Jacques Parienti, Thierry Prazuck, Laure Peyro-Saint-Paul, Anna Fournier, Cécile Valentin, Sylvie Brucato, Renaud Verdon, Aymeric Sève, Mathilda Colin, Fabien Lesne, Jérome Guinard, Meriadeg Ar Gouilh, Julia Dina, Astrid Vabret, Laurent Hocqueloux

**Affiliations:** a Department of Clinical Research and Innovation, Caen University Hospital, Caen, France; b Department of Infectious Diseases, Caen University Hospital, Caen, France; c EA 2656 – Groupe de recherche sur l'adaptation microbienne 2.0, UNICAEN-Université de Caen Normandie, Caen, France; d Department of Infectious Diseases, Orléans Regional Hospital, Orléans, France; e Department of Virology, Orléans Regional Hospital, Orléans, France; f Department of Virology, Caen University Hospital, Caen, France

## Abstract

**Background:**

Tenofovir and emtricitabine interfere with the SARS CoV-2 ribonucleic acid (RNA)-dependent RNA polymerase (RdRp). Several cohorts reported that people treated by tenofovir disoproxil fumarate and emtricitabine are less likely to develop SARS CoV-2 infection and related severe COVID-19.

**Methods:**

We conducted a pilot randomized, open-label, controlled, phase 2 trial at two hospitals in France. Eligible patients were consecutive outpatients (aged ≥18 years) with RT-PCR-confirmed SARS-CoV-2 infection and an interval from symptom onset to enrolment of 7 days or less. Patients were randomly assigned in a 1:1 ratio to receive oral tenofovir disoproxil fumarate and emtricitabine (2 pills on day 1 followed by 1 pill per day on days 2–7) or the standard of care. The primary and secondary endpoints were SARS-CoV-2 viral clearance from baseline assessed by cycle threshold (Ct) RT-PCR on nasopharyngeal swab collected at day 4 and day 7, respectively. A higher Ct corresponds to a lower SARS CoV-2 viral burden. Other endpoints were the time to recovery and the number of adverse events. This trial is registered with ClinicalTrials.gov, NCT04685512.

**Findings:**

From November, 20^th^ 2020 to March, 19^th^ 2021, 60 patients were enrolled and randomly assigned to a treatment group (30 to tenofovir disoproxil fumarate and emtricitabine and 30 to standard of care). The median number of days from symptom onset to inclusion was 4 days (IQR 3–5) in both groups. Amongst patients who received tenofovir disoproxil fumarate, the difference from standard of care in the increase in Ct RT-PCR from baseline was 2.3 (95% confidence interval [-0.6 to 5.2], *p* = 0.13) at day 4 and 2.9 (95% CI [0.1 to 5.2], *p* = 0.044) at day 7. At day 7, 6/30 in the tenofovir disoproxil fumarate and emtricitabine group and 3/30 in the standard of care group reported no COVID-related symptoms. Adverse events included 11 cases of gastrointestinal side effects (grade ≤ 2), three of which leaded to drug discontinuation. Three patients had COVID-19 related hospitalisation, no participant died.

**Interpretation:**

In this pilot study of outpatients adult with recent non-severe COVID-19, tenofovir disoproxil fumarate plus emtricitabine appeared to accelerate the natural clearance of nasopharyngeal SARS-CoV-2 viral burden. These findings support the conduct of larger trials of tenofovir-based therapies for the prevention and early treatment of COVID-19.

**Funding:**

No external funding.

**Research in context**

**Evidence before this study**

Tenofovir and emtricitabine are broad-spectrum antiviral drugs active against HIV and hepatitis B. On June 26th, 2020, del Amo et al. reported that people living with HIV receiving tenofovir disproxil fumarate plus emtricitabine had a lower risk for COVID-19 and related hospitalisation than those receiving other antiretroviral therapies. This has divided the scientific community into two opposing views: a group of reasonably prudent raising the concern of channeling bias, the absence of pre-clinical data and conflicting results from other cohorts; a group of cautiously optimistic calling for investigating this combination in randomized trials. We searched PubMed on June 17th, 2021, using the terms “Covid-19”, “tenofovir”, “random*” for articles in English published up to the date of the search. No randomised controlled trial assessing tenofovir-based therapy in the treatment of patients with COVID-19 was found.

**Added value of this study**

This pilot, randomized, controlled, open-label trial suggests a significant reduction of the SARS-CoV-2 viral burden in nasopharyngeal samples seven days after tenofovir disproxil fumarate plus emtricitabine treatment. This repurposed treatment regimen was also well accepted amongst outpatients with COVID-19, safe, with minor and self-limiting gastrointestinal adverse events, which resolved overtime or upon stopping the medications. Although preliminary, this emerging evidence strengthen the causal relationship regarding the observations reporting a protection of tenofovir-based regimen against COVID-19.

**Implications of all the available evidence**

The positive signal of an antiviral effect against SARS-CoV-2 found in this proof-of-concept study together with evidence of its immunomodulatory effect and other observational studies supports the conduct of larger trials using tenofovir disproxil fumarate plus emtricitabine, alone or in combination with other drugs for the prophylaxis and early treatment of COVID-19, as a global priority level.

Alt-text: Unlabelled box

## Introduction

Coronavirus disease (COVID-19) pandemic incurs public health threat [1]. The global spread of SARS-CoV-2 strikes by its speed and effects, favored by human (large reservoir of susceptible hosts, population density [2], mobility [3]) and viral factors (high degree of contagiousness during the asymptomatic phase [4], increasing affinity to human cells through mutations [5]). Collective efforts focus on the interruption of transmission chains, while hoping for a vaccine-induced or naturally acquired protective herd immunity. Strategies to reduce SARS-CoV-2 community transmission involve individual-level behavior and include face mask [6], [7], social distancing, increase testing, tracing, and isolation. There is no oral “treat” for outpatients COVID-19, yet. Virus-neutralizing monoclonal antibodies demonstrated a significant antiviral effect when administered early in outpatients with COVID-19^7^ and have been granted emergency use in the US and Europe. However, SARS-CoV-2 variants may escape neutralizing antibody responses. [8] In contrast, antivirals are unlikely to be impacted by spike-protein variants. Reduction of the community viral load, as pioneered in the fight against HIV [9], could be critical to decrease SARS CoV-2 basic reproduction number (R0) [10] but effective antivirals that can be prescribed early in the COVID-19 cascade of care by family doctors are lacking. In this line, the experimental drug molnupiravir is currently under investigation, with promising results [11]. In addition to being active against SARS-CoV-2, candidate drugs for an outpatient “test and treat” strategy should be safe including during pregnancy, well tolerated, oral, ideally once-daily [12] and provide individual benefit.

Tenofovir and emtricitabine are both nucleot(s)ide analogues considered essential medicines by the WHO [13] and have been used for decades worldwide as once-daily fixed dose combination to treat HIV, HBV and to prevent HIV infection as pre-exposure prophylaxis. The potential activity of tenofovir against the RNA-dependent RNA polymerase (RdRp) [14], [15], [16], an enzyme indispensable for SARS-CoV-2 replication, has been supported by *in vitro* [17] and *in vivo* [18] experiments. Some observational studies support further evaluation of its use for COVID-19 in humans [19], [20], [21], other do not [22], [23], [24].

We hypothesized that 7-day of tenofovir disoproxil fumarate plus emtricitabine could interfere with SARS-CoV-2 replication in humans. This proof-of-concept study examined the antiviral effect of tenofovir disoproxil fumarate plus emtricitabine on nasopharyngeal viral clearance amongst adults recently SARS-CoV-2 infected with mild-to-moderate COVID-19 not requiring hospitalization.

## Methods

### Study design

The AR0-CORONA (Attenuate R0 Coronavirus) is an investigator-initiated, multicenter, parallel group, open-label, randomized controlled trial assessing the effect of tenofovir disproxil fumarate plus emtricitabine amongst non-hospitalized mild-to-moderate COVID-19 adults recently infected by SARS-CoV-2. Here, we report the preliminary results of the pilot phase 2 trial conducted from November 20^th^, 2020 to March 19^th^, 2021 at two centers in France (Caen University and Orléans Regional hospitals). Outpatients who were tested positive for SARS-CoV-2 for fewer than 5 days at a public or private diagnostic laboratory of the surrounding area of Caen, Normandie and Orléans, Loiret, France were contacted by the investigators for potential participation. The information letter of the protocol was sent by e-mail to potentially interested participants, some of which attended to the investigator site for additional screening. Reasons for nonenrollment were recorded.

Fast-track ethical approval was obtained from the institutional review board of Ile-de-France IV, which covers all site in France (ref 2020/46) on April, 25^th^ 2020. French health ministry approval was obtained from the Agence Nationale de Securité du Médicament (ANSM) on October, 15^th^ 2020 (number EuraCT 2020-001867-94). The trial, which was promoted by Caen University hospital, was conducted in accordance with the principles of the Declaration of Helsinki and the International Conference on Harmonization–Good Clinical Practice guidelines. The study was registered on clintrial.gov (NCT04685512) and the protocol is available online. Written informed consent was obtained from all patients. This report followed the CONSORT guidelines.

### Patients

Eligible patients were consecutive SARS CoV-2 infected adults confirmed by nasopharyngeal swab RT-PCR test and symptoms of COVID-19 for less than 7 days before enrollment, who did not require immediate hospitalization. The protocol allowed for inclusion of asymptomatic patients if the date of SARS-CoV-2 infection was known but this did not occur. Exclusion criteria included: breastfeeding or positive pregnancy test; estimated glomerular filtration rate <80 mL/min per 1.73 m2, as requested by the ANSM; living with HIV, hepatitis B or hepatitis C; contraindication to use the experimental drugs; capillary oximetry less than 95%; clinical evaluation by investigators leading to hospitalisation; use of non-steroid anti-inflammatory or any nephrotoxic drugs during the past 7 days. Patients of child-bearing age (men and women) agreed to take effective contraceptive measures (including hormonal contraception, barrier methods, or abstinence) during the study period. The protocol and data collection took place at a dedicated room at the COVID screening center or at the outpatients consultation of the department of infectious diseases.

### Randomisation and masking

Eligible patients were randomly assigned (1:1) to either the tenofovir disoproxil fumarate plus emtricitabine group or the standard of care group. Computer-generated randomisation list (Enov Clinical®) was centralised and stratified according to the center and the time since the COVID-19 symptoms started (1 to 4 days and 5 to 7 days). The permuted block of size 4 randomisation sequence was prepared by a statistician not involved in the trial using SAS software, version 9.4. After checking eligibility, a trial coordinator assigned participants to a treatment group. We failed to obtain tenofovir disoproxil fumarate plus emtricitabine matching placebo from the pharmaceutical industries and the costs for manufacturing double-blind placebo were prohibitive. Consequently, the study was open-label. The team of virologists who performed SARS CoV-2 RT-PCR was unaware of the treatment group.

### Procedures

At day 1, patients were screened for eligibility; investigators reported medical history, performed clinical and laboratory evaluations and assessed self-reported symptoms related to COVID-19. On the same day 1, eligible patients were assigned to receive either oral pills of 245 mg tenofovir disoproxil fumarate plus 200 mg emtricitabine (as generics, Mylan Laboratories Inc.), as a single tablet regimen, according to the dosage used for on-demand HIV pre-exposure prophylaxis [25] for 7 days (2 pills on day 1 followed by 1 pill on days 2–7) or no treatment aside from usual care. The Caen University hospital pharmacy purchased the tablets (0.8€ per patient) and these were provided free of charge to the patients by dedicated research assistant staff. All participants filled a standard questionnaire assessing self-reported symptoms related to COVID-19, from day 2 to day 3 and from day 5 to day 6. In the treatment group, the questionnaire also assessed adverse events. At day 4 and day 7, participants attended to the clinical site and investigators performed vital signs measurements, clinical examination and assessed self-reported symptoms related to COVID-19. Unannounced plasma tenofovir level was measured at day 4 to check recent drug intake amongst participants in the treatment group. In each of the two participating centers, the same team of trained research nurses performed nasopharyngeal swab for SARS CoV-2 viral RNA detection by RT-PCR assay at day 4 and day 7. The safety assessment included daily monitoring for adverse events, clinical laboratory testing at days 1, 4, and 7 in the treatment group with plasma cell blood count, creatinine, estimated glomerular filtration rate laboratory measurements. Participants were instructed to contact the investigators in case of adverse events or worsening COVID-19 related symptoms. Follow-up ended at 7 days while a phone visit was performed by dedicated research assistants at 14 days to identify later events. Investigators and clinical research assistants in each center performed data collection. Validation, cleaning of the database and data management were done by trial researchers from the Caen University hospital.

### Outcomes

The primary endpoint was the variation of SARS-CoV2 viral load burden from baseline assessed by the variation of the number of cycle thresholds (Ct) of RT-PCR from nasopharyngeal samples at day-4 compared to baseline. Secondary outcome was the variation of the number of cycle thresholds (Ct) of RT-PCR at day-7, corresponding to the end of treatment, compared to baseline. RT-PCR assay was performed on fresh samples in each participating center. A *post-hoc* sensitivity analysis used frozen samples from one center for conducting centralized RT-PCR. Details of the RT-PCR methods are described in the Supplemental Appendix. A decrease of SARS-CoV2 viral load corresponded to an increase in Ct.

Other outcomes were the number and grade of adverse events according to the Common Terminology Criteria for Adverse Events version 5.0, serious adverse events, premature discontinuations of study drug, the time to complete resolution of COVID-19 related symptoms, hospitalization and death. Resolution of symptoms was assessed sequentially using the daily symptoms questionnaire and recorded information on the presence and type of symptoms; complete resolution endpoint was reached when no COVID-19– related symptoms was reported.

### Statistical analysis

We estimated that a sample size of 60 patients would provide the trial with 85% power to detect a difference of 4 Ct in the mean reduction of RT-PCR SARS-CoV-2 viral load at day 4 with a 1-sided significance level of α = 0.025, assuming an expected standard deviation (SD) of 5. Considering the uncertainty regarding the efficacy of tenofovir disoproxil fumarate plus emtricitabine in reducing RT-PCR SARS-CoV-2 viral load, we planned one interim analysis after enrollement of 30 subjects according to an O'Brien Fleming design with stopping rules for both: efficacy (*p* < 0.00516) and futility (*p* > 0.544). None of these boundaries was crossed and the full sample size of the phase 2 was reached.

The primary efficacy analysis was performed on the intention-to-treat (ITT) population, which included all randomized participants analyzed in their assignment group. Quantitative data were checked for normal distribution by Kolmogorov-Smirnov test. Baseline characteristics were described by means (standard deviation), median [interquartile range] and numbers (percentages), as appropriate. Regarding the primary outcome, the RT-PCR SARS-CoV-2 Ct variation from baseline to day 4 (day-4 less baseline) was compared between groups by an analysis of covariance (ANCOVA) including the treatment group as fixed and adjusting for design factors used to stratify the randomization (center and prior duration of symptoms), as appropriate. The same strategy was used for day 7. Pre-planned subgroup analyses were performed for factors used in the randomization scheme using appropriate ANCOVA models. Qualitative secondary outcomes were compared numerically. The time to resolution of symptoms was plotted with Kaplan-Meier curves stratified by treatment group and compared by the Log-rank test. Laboratory safety measures in the treatment group overtime were compared to baseline by the Student *t*-test for paired samples. All statistical analyses were computed with SAS software V9.4 (SAS institute, NC, Cary). A p-value of less than 0.048 was considered significant for the primary outcome to take into account the interim analysis and other statistical testing were exploratory.

### Role of the funding

The funder of the study had no role in study design, data collection, data analysis, data interpretation, or writing of the report. The first and last authors had full access to all the data in the study and had final responsibility for the decision to submit for publication.

## Results

**Fig. 1:**
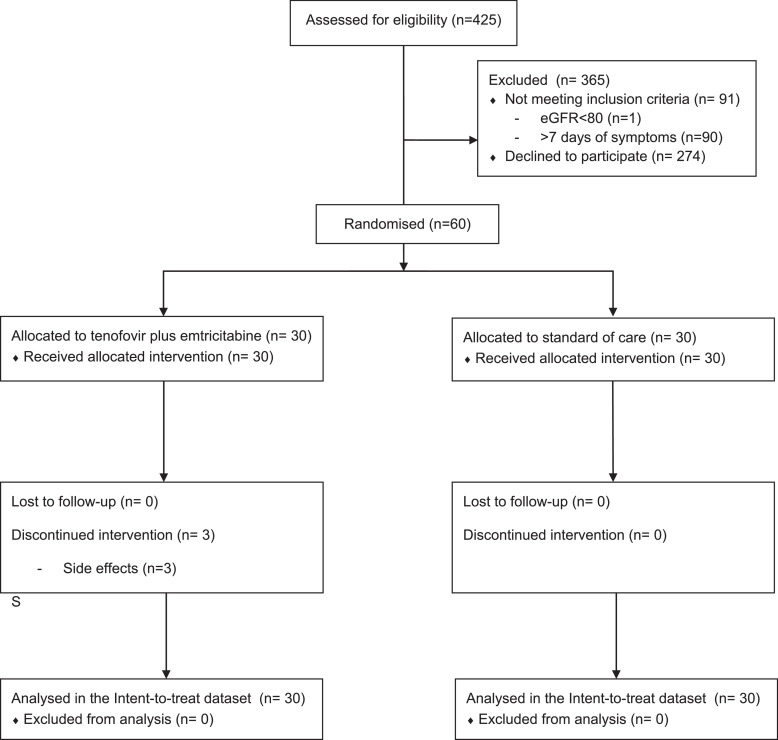
Flow diagram of participant selection and allocation.

Of 425 outpatients with SARS-CoV-2 positive RT-PCR approached between November 20^th^, 2020 to March 19^th^ 2021, at the two investigator sites, 60 (14%) were consecutively enrolled (Fig. 1): 30 were assigned to the tenofovir disoproxil fumarate plus emtricitabine group and 30 to the standard of care group. Non-inclusion was mainly due to not being interested and non-eligibility due to symptoms starting 7 days prior to possible inclusion. One participant failed screening because of an estimated glomerular filtration rate <80 mL/min per 1.73 m2. All participants attended to the visits; there was no loss of follow-up.

Baseline characteristics (Table 1) were well balanced between the two allocation groups. Median age was 42, the majority of the patients were women (57%) and comorbidities were rare. All had COVID-19 related symptoms at baseline, fatigue being the most reported amongst a median of 5 reported symptoms. The median number of days from symptom onset to inclusion was 4 days (IQR 3–5) in both groups. No other potential active drug was administered to the participants.

**Table 1:** Baseline participant characteristics by randomized groups

	TDF/FTC (n = 30)	Standard of Care (n = 30)
Mean Age (SD), year, mean (SD)	39.9 (14.8)	42.6 (16.7)
Women	16 (53%)	18 (60%)
Body-mass index, kg/m^2^	24.9 (3.7)	23.7 (3.7)
Health care worker	17 (57%)	18 (60%)
Smoker	8 (27%)	10 (33%)
Comorbid condition		
Hypertension	2 (7%)	1 (3%)
Diabetes	1 (3%)	1 (3%)
Moderate COVID-19 (vs mild)	1 (3%)	2 (6%)
Duration of prior symptoms, median [IQR]	4 [3-5]	4 [3-5]
2 or <2 d	6 (20%)	7 (23%)
3-4 d	14 (47%)	15 (50%)
>4 d	10 (33%)	8 (27%)
Symptoms at baseline	30 (100%)	30 (100%)
Olfactory loss	15 (50%)	12 (40%)
Gustatory loss	15 (50%)	10 (33%)
Fatigue	20 (67%)	24 (80%)
Cough	16 (53%)	18 (60%)
Headache	17 (57%)	16 (53%)
Shortness of breath	11 (37%)	9 (30%)
Nausea	5 (17%)	8 (27%)
Rhinitis	14 (47%)	20 (67%)
Number of symptoms at baseline, median [IQR]	5 [3–8]	5 [4–6]
Vital signs		
Respiratory frequency, cycle/min	17.8 (2.7)	17.1 (2.6)
Oxymetry, (%)	98.7 (1.3)	98.8 (1.2)
Temperature,°C	36.8 (0.9)	36.7 (0.8)
Heart rate, beat/min	77 (15)	80 (13)
Laboratory measures		
SARS-CoV-2 Ct RT-PCR per swab, mean (SD)	21.0 (5.3)	23.5 (5.5)
e-GFR, mL/min, mean (SD)	122 (30)	111 (31)
C-reactive protein, mg/L, mean (SD)*	6.8 (8.8)	6.4 (8.2)
<5 mg/L	18 (60%)	22 (73%)
5 to 10 mg/mL	8 (27%)	3 (10%)
>10 mg/mL	4 (13%)	5 (17%)

*Data missing for 2 patients; TDF/FTC, tenofovir disoproxil fumarate and emtricitabine; IQR, interquartile range; SD, Standard deviation.

None of the differences between groups was statistically significant.

The Fig. 2 and Table 2 display the virologic outcomes at day 4 and day 7. Amongst patients who received tenofovir disoproxil fumarate, the difference from standard of care in the increase in Ct RT-PCR from baseline was 2.3 (95% confidence interval [-0.6 to 5.2], *p* = 0.13) at day 4 and 2.9 (95% CI [0.1 to 5.2], *p =* 0.044) at day 7. This effect was homogeneous within subgroups stratified by center and duration of symptoms (Table 2). Because a high Ct is a surrogate marker of a lower viral load, our results show a higher decrease of the SARS-CoV-2 viral burden amongst treated participants. These results were consistent with the sensitivity analysis presented in Supplemental Figure 1.

**Figure:**
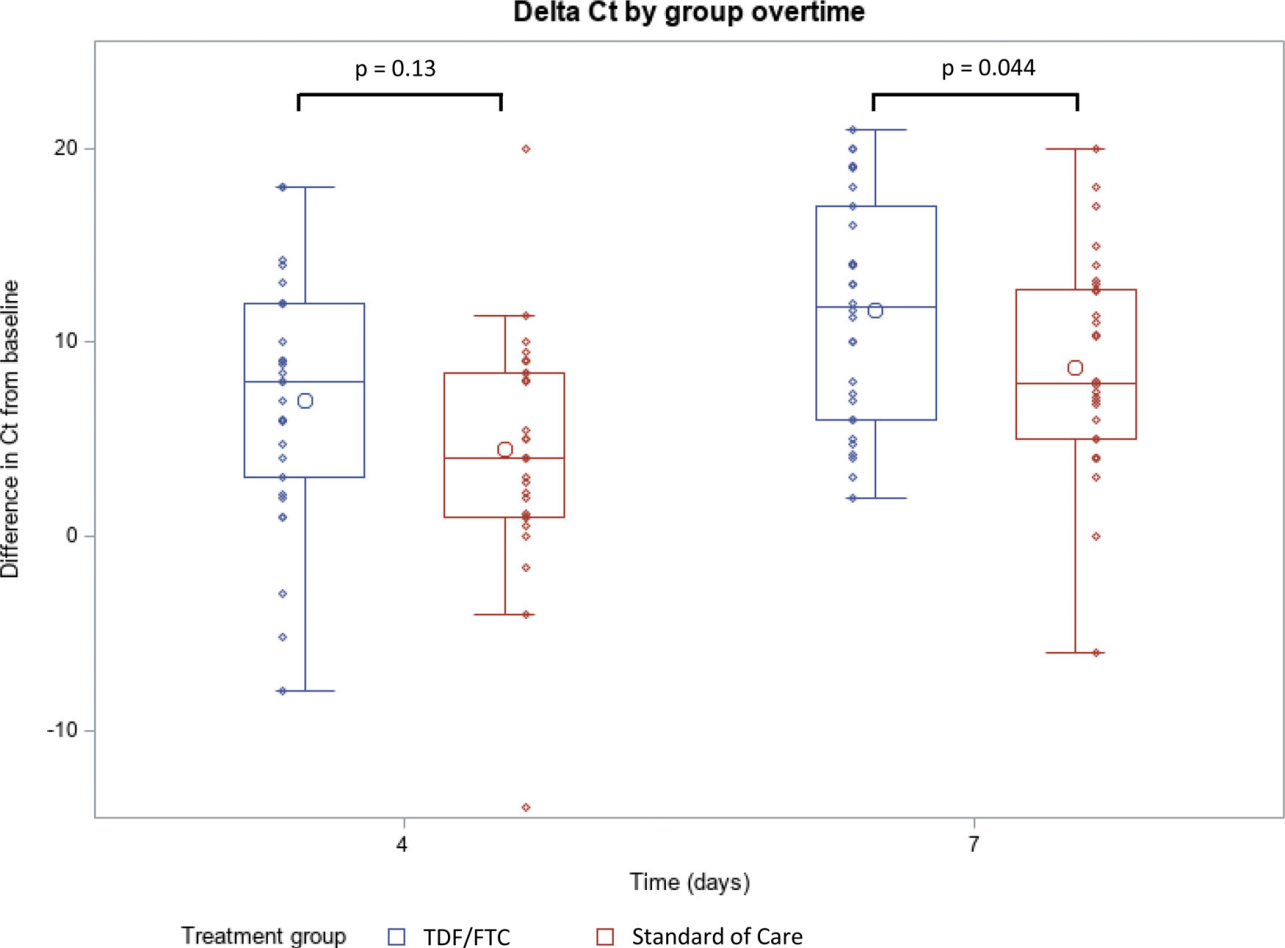
Fig. 2. Variation of Ct RT-PCR for SARS-CoV-2 in fresh nasopharyngeal samples by study visit according to allocated groups. The length of the box represents the interquartile range (IQR, the distance between the 25^th^ and 75^th^ percentiles). The horizontal line and the larger circle within the box are the median and the arithmetic mean, respectively. The upper and lower whiskers mark the more extreme observations within 1.5(IQR) above the 75th percentiles and within 1.5(IQR) below the 25th percentiles, respectively. The smaller circles are the raw data.

**Table 2:** Effect of Tenofovir Disproxil Fumarate Plus Emtricitabine (TDF/FTC) on outcomes

	TDF/FTC (n = 30)	Standard of Care (n = 30)	Difference (95% CI)	p-value
Primary outcome				
Variation of Ct from baseline to day 4	7.0 (6.2)	4.4 (5.9)	2.3 [-0.6 to 5.2]*	0.13*
Center 1, n = 39			3.0 [-1.0 to 6.9]¥	
Center 2, n = 21			0.9 [-3.0 to 4.8]¥	
Symptoms ≤ 4 days, n = 42			1.7 [-2.0 to 5.5]¥	
Symptoms > 4 days, n = 18			3.5 [-0.6 to 7.7]¥	
Secondary outcome				
Variation of Ct from baseline to day 7	11.6 (5.9)	8.7 (5.6)	2.9 [0.4 to 5.2]*	0.044*
Center 1, n = 39			3.1 [-0.9 to 7.0]¥	
Center 2, n = 21			2.6 [-0.8 to 6.1]¥	
Symptoms ≤ 4 days, n = = 42			2.5 [-1.1 to 6.1]¥	
Symptoms > 4 days, n = 18			4.0 [-0.2 to 8.2]¥	
Other outcomes				
Detectable plasma TFV at day 4	30 (100%)	-	-	-
Plasma TFV level at day 4, mean (SD)	117 (129)	-	-	-
No COVID-related symptoms by day 7	6 (20%)**	3 (10%)**	-	0.29**
Any adverse event				
Grade 1	6 (20%)	NA		
Grade 2	5 (17%)	NA		
Grade 3 or more	0	NA		
Hospitalization (serious adverse event)	2 (6%)	1 (3%)	-	-
Death	0	0	-	-

*Generalized linear model adjusting for duration of symptoms and center

** Kaplan-Meir estimates compared by the Log-rang test

NA: not applicable due to the open label design

TFV: tenofovir, ng/mL; CI, confidence interval

¥ Generalized linear model adjusting for duration of symptoms or center, as appropriate

**Fig. 3:**
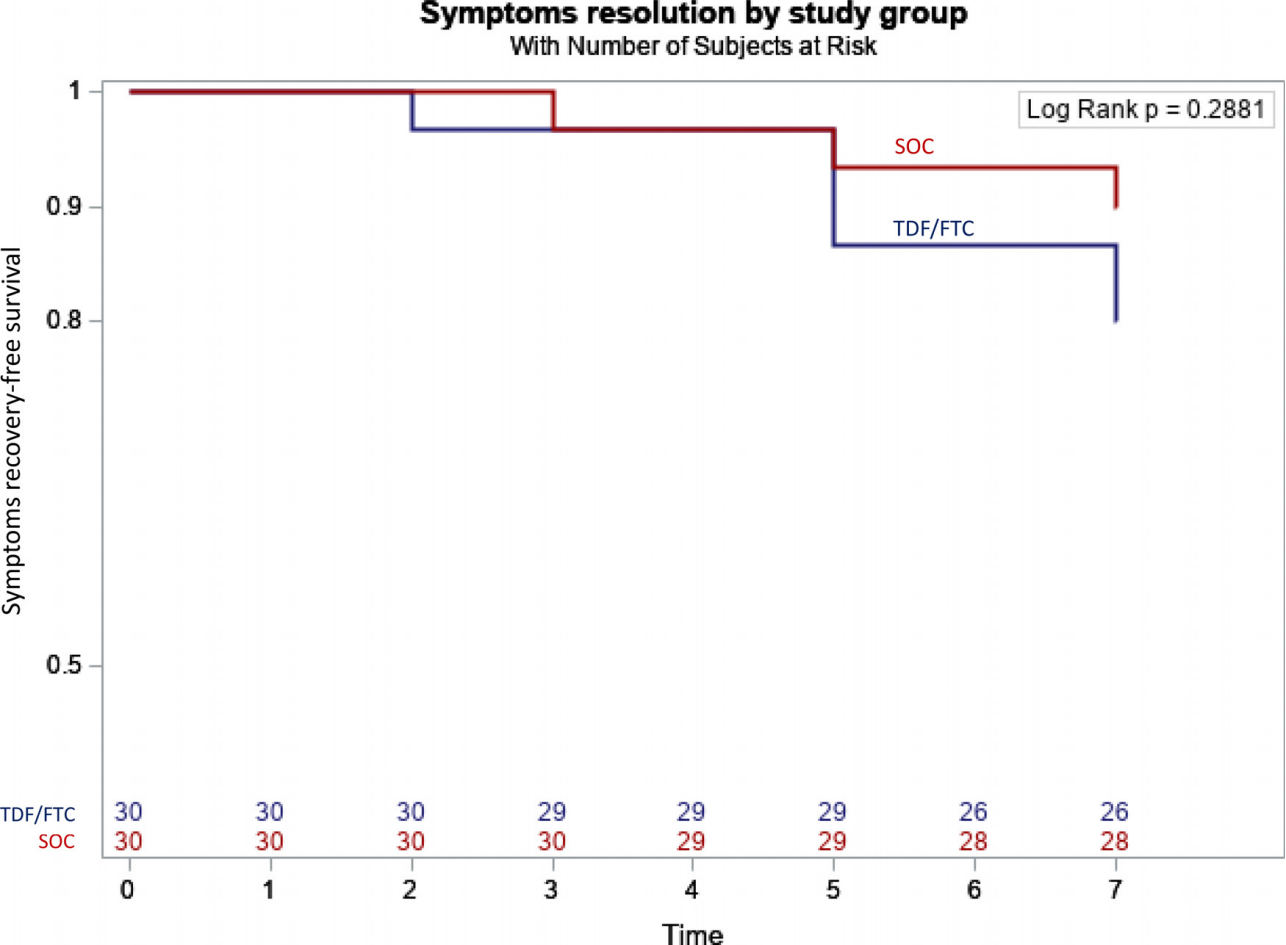
Kaplan-Meier curves of symptomatic patients according to allocated groups

The Fig. 3 shows Kaplan-Meier curves representing the proportion of patients who self-reported at least one COVID-related symptom during the study. The majority of participant were still experiencing symptoms at day 7. The time to symptoms recovery was similar between groups (*p =* 0.29). More specifically, 6/30 in the tenofovir disoproxil fumarate and emtricitabine group and 3/30 in the standard of care group had no COVID-related symptoms by day 7.

All plasma tenofovir levels were above the limit of detection, suggesting exposure to the experimental drug prior to day 4 visit (Table 2) amongst participants assigned to tenofovir disoproxil fumarate plus emtricitabine. In the treatment group, the mean (SD) variation of eGFR were -2.8 mL/min per 1.73 m2 (95% confidence interval [-10.2 to 4.5], *p =* 0.43) from baseline to day 4 and -2.8 mL/min per 1.73 m2 (95% confidence interval [-9.0 to 3.4], *p =* 0.36) from baseline to day 7. Two men (14% among 14) and 9 women (56% among 16) reported nausea or vomiting (grade ≤ 2), three of which leaded to drug discontinuation after day-4. Three serious adverse events, not related to the study drugs according to the investigators, occurred. In the tenofovir disoproxil fumarate plus emtricitabine, a 60 years old woman without co-morbidity included at the first visit with dypnea (22 breath/min), very high RT-PCT viral load (14 Ct) and inflammatory syndrome (CRP = 21 mg/L) was hospitalised at day 7 (28 Ct) for non-severe COVID-related pneumonia requiring oxygen (2 L/min) which recovered. In the tenofovir disoproxil fumarate plus emtricitabine, a 68 years old woman without co-morbidity included at the first visit with fever (39.1°C), moderate RT-PCT viral load (28 Ct) was hospitalised at day 7 (35 Ct) for severe COVID-related pneumonia requiring high flow oxygen, which recovered without mechanical ventilation. In the standard of care group, a 64 years old man with diabete and hypertension included at the first visit with fever (38.7°C), high RT-PCT viral load (18 Ct) and inflammatory syndrome (CRP = 18.6 mg/L) was admitted at day 7 (20 Ct) for severe COVID-related pneumonia requiring oxygen (6 L/min) which recovered. No patients died during the study.

## Discussion

This proof-of-concept randomized clinical trial suggests, for the first time in man, the potential efficacy of the tenofovir disoproxil fumarate plus emtricitabine combination in reducing the amount of virus present in the nasopharyngeal secretions of adults symptomatic of COVID-19. This effect was not significant on day 4, which was the primary endpoint, but later on day 7. This result, although preliminary, supports the value of examining the repositioning of this treatment as an additional tool capable of reducing the SARS-CoV-2 viral load including transmission of the virus from an index case.

As large as 64% of the potentially eligible patients declined to participate at the initial phone assessment. This rate contrasts with another study testing hydroxychloroquine in this setting (11% in [26]). Several factors may explain our low rate of participation: the good prognostic of non-severe COVID-19, the inconvenience of additional visits and testing, the fear of taking HIV drugs (and associated side-effects) and the fact that tenofovir disoproxil fumarate plus emtricitabine received no media coverage at the time our study was recruiting, in contrast with hydroxychloroquine. In addition, a disproportional rate of healthcare workers (58%) were enrolled, which is consistent with other trials (86% in [26]; 57% in [27]). This might reflect the proximity to the investigator site, the confidence in clinical research and a lower fear of side effects.

Tenofovir disoproxil fumarate plus emtricitabine decreased (an effect corresponding to approximately 0.8 log_10_ decrease of SARS CoV-2 RNA) the viral load burden on day-7 compared to the standard of care. Of note, the WHO Working Group on the Clinical Characterisation and Management of COVID-19 infection recommended this surrogate endpoint to measure pathogen burden in response to treatment [28]. Mitjà and collaborators [26] who investigated hydroxychloroquine in a similar COVID-19 population suggested a window of at least 7 days for viral load reduction assessment and an effect size of at least 0.5 log_10_ reduction.

During the first week of COVID-19, known as the “viral” phase of the disease, the replication peaks in the upper airways. This phase is responsible for the transmissibility of the virus [29] first and followed by COVID-related symptoms. A better clearance of SARS-CoV-2 viral RNA on day 7 has been associated with a decreased risk of hospitalization in a randomized trial investigating neutralizing antibody LY-CoV555 amongst outpatients with mild-to-moderate COVID-19^7^. In addition, high levels of SARS-CoV-2 viral load (≥6 log_10_ copies per mL) at days 7 post symptom onset was significantly associated with mortality in a cohort of 284 hospitalized COVID-19 patients [30].

The new results reported here could shed more light on the observational associations between tenofovir disoproxil fumarate plus emtricitabine use, COVID-19 risk of infection, and COVID-19 related outcomes. In a cohort of 77,590 people living with HIV in Spain, del Amo and collaborators [19] reported a protective effect of tenofovir disoproxil fumarate and emtricitabine compared to other antiretroviral therapies in reducing the risk for COVID-19 diagnosis and hospitalization. Acknowledging the large sample size and the magnitude of this association, chanelling bias was a reasonable alternative interpretation because younger patients with no co-morbidity are more likely to: (i) receive tenofovir disoproxil fumarate (ii) have a good COVID-19 prognosis. Noteworthy, these findings were robust to stratification on age and instrumental variable analyses [31]. In a cohort study of 750 men who have sex with men and transgender women also from Spain [32], PrEP users who tested positive for SARS-CoV-2 antibodies showed twice more asymptomatic infection (42.7% of PrEP users vs. 21.7% of non-PrEP users; *p =* 0.07). In the Spanish HIV Research Network Cohort, tenofovir disoproxil fumarate plus emtricitabine therapy was independently associated with a 3-fold lower prevalence of COVID-19 seropositivity (5/154 (3.2%) versus 40/416 (9.6%) with tenofovir alafenamide therapy, adjusted Odds Ratio, 0.32, 95% confidence interval (0.12 to 0.84); *p =* .021) controlling for age, sex and comorbidities [21]. Finally, people living with HIV in South Africa with documented antiretrovirals dispensed in the last 12 months who contracted COVID-19 were less likely to die (adjusted hazard ratio, 0.41, 95% confidence interval (0.21 to 0.78), *p =* 0.007) if they received tenofovir disoproxil fumarate and emtricitabine rather than other antiretrovirals, controlling for age, sex and comorbidities [20]. For comparison, the adjusted hazard ratios for death were 0.56 (95% confidence interval (0.47 to 0.68)) for 1 dose of BNT162b2, 0.45 (95% confidence interval (0.34 to 0.59)) for 1 dose of ChAdOx1 and 0.31 (95% confidence interval 0.14 to 0.69) for 2 doses of BNT162b2, compared to unvaccinated cases while controlling for age, sex and comorbidities [33].

The absence of positive signal between tenofovir alafenamide treatment[ 19, 21] and COVID-19 outcome is intriguing because tenofovir disoproxil fumarate and tenofovir alafemanide both yield to the same active compound, namely tenofovir. While the former concentrates in plasma, the latter concentrates in the cells, in particular lymphatic cells [34]. These pharmacokinetics differences may provide an advantage for tenofovir disoproxil fumarate in reaching SARS-CoV-2 infected cells, in particular the endothelium [35] and highly vascularized organs such as the kidneys or the lungs. Tenofovir disoproxil fumarate and tenofovir alafenamide have a very different lipid metabolism and lipids play a fundamental role in viral replication [36]. Finally, tenofovir disoproxil fumarate has immunomodulatory effects[ 37, 38], which could be of importance in limiting the cytokine storm during the second week of COVID-19, known as the "inflammatory" phase of the disease. For example, the administration of tenofovir disoproxil fumarate plus emtricitabine for 30 days was associated with a significant decrease of HLA-DR+ CD38+ co-expression on CD8+ T-cells, a marker of immune activation amongst 19 healthy volunteers [39]. Of note, HLA-DR+ CD38+ co-expression on CD8+ T-cells was significantly associated with COVID-19 severity in a cohort of 125 COVID-19 patients [40].

Overall, tenofovir disoproxil fumarate plus emtricitabine was well tolerated, as expected for a short course of 7 days. The mild to moderate gastrointestinal adverse events observed in this study were similar to those previously reported (10%) with on-demand PrEP [25] among men, but with a higher frequency in our trial using PrEP like dosing. Of note, women included in COVID-19 trials were more likely to report adverse events [41] and more than half of our participants were women.

We are aware of limitations. Like any proof-of-concept study, the sample size was small. Several factors, such as age [30] could influence the SARS-CoV-2 viral load kinetics. Despite stratified randomization on the duration of symptoms prior to inclusion, these or other unknown factors may cause residual confounding. Before embarking in a larger trial involving several centers, this pilot phase 2 trial was designed to explore the feasibility and potential signal for the use of tenofovir disoproxil fumarate plus emtricitabine in outpatients COVID-19. We tested the combination of tenofovir disoproxil fumarate plus emtricitabine. We cannot conclude on the efficacy of each drug separately. Our choice was dictated by the excellent safety profile of emtricitabine and the pre-clinical data available, conducted with this fixed-dose regimen [18]. We did not collect pharyngeal swab for RT-PCR at the time of inclusion. Therefore, any decrease in viral load burden between baseline and inclusion was not appreciated. Inclusion was restricted to outpatients treated early in the course of non-severe but symptomatic COVID-19. Therefore, our results may not apply to later COVID-19 stages. A mean reduction of 2.9 Ct is of unknown microbiological or clinical relevance and patients had no individual benefit. However, our study was neither designed nor powered to investigate clinical outcome. Finally, while our primary endpoint was objective, the open label design did not control for the placebo effect, in particular the potential for self-reporting bias in the data reporting COVID-19-related symptoms by questionnaire. In addition, because the digestive symptoms of COVID-19 might mimic tenofovir disoproxil fumarate plus emtricitabine adverse events, the assessment of clinical tolerance and clinical resolution of symptoms may be biased.

This work has also strengths. It is the first to support in a clinical trial an antiviral effect of tenofovir disoproxil fumarate, an overlooked potential repurposing drug with a structure similar to remdesivir, but compatible with outpatient COVID-19 treatment. The statistically significant results despite the small sample size (60 subjects) together with the immunomodulatory properties [37], [38], [39] of tenofovir disoproxil fumarate may be the promise of a clinically important effect as supported by the accumulating observations reporting a protection of tenofovir disoproxil fumarate plus emtricitabine against COVID-19 [19], [20], [32], [42]. The investigated drugs are in the public domain, which increases the potential of their use in low-ressource countries, also affected by the COVID-19 but with lower access to vaccine or potential new antivirals [11]. As stated by DeJong et al [43]: “*Importantly, even if tenofovir has lower efficacy than other experimental agents against COVID-19, its wide availability and low cost might still enable a greater public health impact.”* Finally, the long lasting experience of using this combination is reassuring, including during pregnancy [44].

In conclusion, tenofovir disoproxil fumarate plus emtricitabine significantly decreased the SARS-CoV-2 viral burden in recently infected COVID-19 outpatients. Although time-limited exposure to tenofovir disoproxil fumarate plus emtricitabine had a good safety profile, we do not advocate its experimental use in clinical practice based on this proof-of-concept study. However, this pilot randomized clinical trial moves the rational of trials investigating tenofovir disoproxil fumarate plus emtricitabine for COVID-19 prevention (NCT04334928) and treatment (NCT04712357; NCT04359095; NCT04890626) currently recruiting one step-forward.

## Declaration of Competing Interest

J.-JP reports personal fees and grant support from Gilead Sciences, Merck Sharp & Dohme and ViiV Healthcare outside the submitted work. L.H. reports personal fees and non-financial support from Gilead Sciences, Janssen-Cilag, Merck Sharp & Dohme and ViiV Healthcare outside the submitted work. Other authors declare no competing interests.

## Appendix Supplementary materials

Image, application 1

## References

[1] COVID-19 Map. Johns Hopkins Coronavirus Resource Center. https://coronavirus.jhu.edu/map.html (accessed March 29, 2021).

[2] Wong DWS, Li Y. Spreading of COVID-19: Density matters. *PLoS One*2020; 15: e0242398. 10.1371/journal.pone.0242398 PMC7757878 33362283

[3] Phan LT, Nguyen TV, Luong QC, et al. Importation and human-to-human transmission of a Novel Coronavirus in Vietnam. *N Engl J Med*2020; 382: 872–4. 10.1056/NEJMc2001272 PMC7121428 31991079

[4] Arons MM, Hatfield KM, Reddy SC, et al. Presymptomatic SARS-CoV-2 infections and transmission in a skilled nursing facility. *New England Journal of Medicine*2020; published online April 24. DOI:10.1056/NEJMoa2008457. 10.1056/NEJMoa2008457 PMC7200056 32329971

[5] Starr TN, Greaney AJ, Hilton SK, et al. Deep Mutational Scanning of SARS-CoV-2 receptor binding domain reveals constraints on folding and ACE2 Binding. *Cell*2020; 182: 1295-1310.e20. 10.1016/j.cell.2020.08.012 PMC7418704 32841599

[6] Gandhi M, Rutherford GW. Facial masking for COVID-19 — Potential for “Variolation” as we await a vaccine. *New England Journal of Medicine*2020; published online Sept 8. DOI:10.1056/NEJMp2026913. 10.1056/NEJMp2026913 PMC7890559 32897661

[7] Chen P, Nirula A, Heller B, et al. SARS-CoV-2 Neutralizing Antibody LY-CoV555 in Outpatients with Covid-19. *N Engl J Med*2021; 384: 229–37. 10.1056/NEJMoa2029849 PMC7646625 33113295

[8] Hoffmann M, Arora P, Groß R, et al. SARS-CoV-2 variants B.1.351 and P.1 escape from neutralizing antibodies. *Cell*2021; published online March 20. DOI:10.1016/j.cell.2021.03.036. 10.1016/j.cell.2021.03.036 PMC7980144 33794143

[9] Das M, Chu PL, Santos G-M, et al. Decreases in community viral load are accompanied by reductions in new HIV infections in San Francisco. *PLoS ONE*2010; 5: e11068. 10.1371/journal.pone.0011068 PMC2883572 20548786

[10] Dietz K. The estimation of the basic reproduction number for infectious diseases. *Stat Methods Med Res*1993; 2: 23–41. 10.1177/096228029300200103 8261248

[11] Painter W-P, Sheahan T, Baric R, et al. Reduction in infectious SARS-CoV-2 in treatment study of COVID-19 with MOLNUPIRAVIR. CROI Conference. https://www.croiconference.org/abstract/reduction-in-infectious-sars-cov-2-in-treatment-study-of-covid-19-with-molnupiravir/ (accessed May 18, 2021).

[12] Parienti J-J, Bangsberg DR, Verdon R, Gardner EM. Better adherence with once-daily antiretroviral regimens: a meta-analysis. *Clin Infect Dis*2009; 48: 484–8. 10.1086/596482 PMC2708315 19140758

[13] WHO model list of essential medicines. https://www.who.int/publications-detail-redirect/WHOMVPEMPIAU2019.06 (accessed March 29, 2021).

[14] Elfiky AA. Ribavirin, Remdesivir, Sofosbuvir, Galidesivir, and Tenofovir against SARS-CoV-2 RNA dependent RNA polymerase (RdRp): A molecular docking study. *Life Sci*2020; 253: 117592. 10.1016/j.lfs.2020.117592 PMC7102646 32222463

[15] Chien M, Anderson TK, Jockusch S, et al. Nucleotide analogues as inhibitors of SARS-CoV-2 Polymerase, a Key Drug Target for COVID-19. *J Proteome Res*2020; 19: 4690–7. 10.1021/acs.jproteome.0c00392 PMC7640960 32692185

[16] Zandi K, Amblard F, Musall K, et al. Repurposing nucleoside analogs for human coronaviruses. *Antimicrob Agents Chemother*2020; 65. DOI:10.1128/AAC.01652-20. 10.1128/AAC.01652-20 PMC7927863 33122172

[17] Clososki GC, Soldi RA, Silva RM da, et al. Tenofovir Disoproxil Fumarate: New chemical developments and encouraging in vitro biological results for SARS-CoV-2. *J Braz Chem Soc*2020; 31: 1552–6.

[18] Park S-J, Yu K-M, Kim Y-I, et al. Antiviral Efficacies of FDA-approved drugs against SARS-CoV-2 infection in Ferrets. *mBio*2020; 11. DOI:10.1128/mBio.01114-20. 10.1128/mBio.01114-20 PMC7244896 32444382

[19] Del Amo J, Polo R, Moreno S, et al. Incidence and Severity of COVID-19 in HIV-positive persons receiving antiretroviral therapy : a cohort study. *Ann Intern Med*2020; 173: 536–41. 10.7326/M20-3689 PMC7394316 32589451

[20] Boulle A, Davies M-A, Hussey H, et al. Risk factors for COVID-19 death in a population cohort study from the Western Cape Province, South Africa. *Clin Infect Dis*2020; published online Aug 29. DOI:10.1093/cid/ciaa1198. 10.1093/cid/ciaa1198 PMC7499501 32860699

[21] Berenguer J, Diez C, Martin-Vincente M, et al. Prevalence and factors associated with SARS-CoV-2 antibodies in a SPANISH HIV Cohort. CROI Conference. https://www.croiconference.org/abstract/prevalence-and-factors-associated-with-sars-cov-2-antibodies-in-a-spanish-hiv-cohort/ (accessed March 30, 2021).

[22] Härter G, Spinner CD, Roider J, et al. COVID-19 in people living with human immunodeficiency virus: a case series of 33 patients. *Infection*2020; 48: 681–6. 10.1007/s15010-020-01438-z PMC7211976 32394344

[23] Gudipati S, Brar I, Murray S, McKinnon JE, Yared N, Markowitz N. Descriptive analysis of patients living with HIV affected by COVID-19. *J Acquir Immune Defic Syndr*2020; 85: 123–6. 10.1097/QAI.0000000000002450 PMC7446990 32675771

[24] Isernia V, Julia Z, Le Gac S, et al. SARS-COV2 infection in 30 HIV-infected patients followed-up in a French University Hospital. *Int J Infect Dis*2020; 101: 49–51. 10.1016/j.ijid.2020.09.1436 PMC7518976 32987182

[25] Molina J-M, Capitant C, Spire B, et al. On-demand preexposure prophylaxis in men at high risk for HIV-1 infection. *N Engl J Med*2015; 373: 2237–46. 10.1056/NEJMoa1506273 26624850

[26] Mitjà O, Corbacho-Monné M, Ubals M, et al. Hydroxychloroquine for early treatment of adults with mild Covid-19: a randomized-controlled trial. *Clin Infect Dis*2020; published online July 16. DOI:10.1093/cid/ciaa1009. 10.1093/cid/ciaa1009 PMC7454406 32674126

[27] Skipper CP, Pastick KA, Engen NW, et al. Hydroxychloroquine in non-hospitalized adults with early covid-19 : a randomized trial. *Ann Intern Med*2020; 173: 623–31. 10.7326/M20-4207 PMC7384270 32673060

[28] WHO Working Group on the Clinical Characterisation and Management of COVID-19 infection. a minimal common outcome measure set for COVID-19 clinical research. *Lancet Infect Dis*2020; 20: e192–7. 10.1016/S1473-3099(20)30483-7 PMC7292605 32539990

[29] Gandhi RT, Lynch JB, Del Rio C. Mild or moderate Covid-19. *N Engl J Med*2020; 383: 1757–66. 10.1056/NEJMcp2009249 32329974

[30] Néant N, Lingas G, Le Hingrat Q, et al. Modeling SARS-CoV-2 viral kinetics and association with mortality in hospitalized patients from the French COVID cohort. *Proc Natl Acad Sci U S A*2021; 118. DOI:10.1073/pnas.2017962118. 10.1073/pnas.2017962118 PMC7929555 33536313

[31] Del Amo J, Polo R, Moreno S, et al. Antiretrovirals and risk of COVID-19 diagnosis and hospitalization in HIV-positive persons. *Epidemiology*2020; 31: e49–51. 10.1097/EDE.0000000000001235 PMC7541717 32773469

[32] Ayerdi O, Puerta T, Clavo P, et al. Preventive Efficacy of Tenofovir/Emtricitabine against severe acute respiratory syndrome coronavirus 2 Among Pre-Exposure Prophylaxis Users. *Open Forum Infect Dis*2020; 7: ofaa455. 10.1093/ofid/ofaa455 PMC7543639 33200081

[33] Bernal JL, Andrews N, Gower C, et al. Effectiveness of BNT162b2 mRNA vaccine and ChAdOx1 adenovirus vector vaccine on mortality following COVID-19. *medRxiv*2021; 2021.05.14.21257218.

[34] Lee WA, He G-X, Eisenberg E, et al. Selective intracellular activation of a novel prodrug of the human immunodeficiency virus reverse transcriptase inhibitor tenofovir leads to preferential distribution and accumulation in lymphatic tissue. *Antimicrob Agents Chemother*2005; 49: 1898–906. 10.1128/AAC.49.5.1898-1906.2005 PMC1087627 15855512

[35] Varga Z, Flammer AJ, Steiger P, et al. Endothelial cell infection and endotheliitis in COVID-19. *Lancet*2020; 395: 1417–8. 10.1016/S0140-6736(20)30937-5 PMC7172722 32325026

[36] Lorizate M, Kräusslich H-G. Role of lipids in virus replication. *Cold Spring Harb Perspect Biol*2011; 3: a004820. 10.1101/cshperspect.a004820 PMC3179339 21628428

[37] Melchjorsen J, Risør MW, Søgaard OS, et al. Tenofovir selectively regulates production of inflammatory cytokines and shifts the IL-12/IL-10 balance in human primary cells. *J Acquir Immune Defic Syndr*2011; 57: 265–75. 10.1097/QAI.0b013e3182185276 21471820

[38] Zídek Z, Franková D, Holý A. Activation by 9-(R)-[2-(phosphonomethoxy)propyl]adenine of chemokine (RANTES, macrophage inflammatory protein 1alpha) and cytokine (tumor necrosis factor alpha, interleukin-10 [IL-10], IL-1beta) production. *Antimicrob Agents Chemother*2001; 45: 3381–6. 10.1128/AAC.45.12.3381-3386.2001 PMC90841 11709312

[39] Castillo-Mancilla JR, Meditz A, Wilson C, et al. Reduced immune activation during tenofovir-emtricitabine therapy in HIV-negative individuals. *J Acquir Immune Defic Syndr*2015; 68: 495–501. 10.1097/QAI.0000000000000529 PMC4358752 25763783

[40] Mathew D, Giles JR, Baxter AE, et al. Deep immune profiling of COVID-19 patients reveals distinct immunotypes with therapeutic implications. *Science*2020; 369. DOI:10.1126/science.abc8511. 10.1126/science.abc8511 PMC7402624 32669297

[41] Zekarias A, Watson S, Vidlin SH, Grundmark B. Sex differences in reported adverse drug reactions to COVID-19 drugs in a global database of individual case safety reports. *Drug Saf*2020; 43: 1309–14. 10.1007/s40264-020-01000-8 PMC7518652 32978702

[42] Cornejo-Giraldo M, Rosado N, Salinas J, et al. Tenofovir-DF versus Hydroxychloroquine in the treatment of hospitalized patients with COVID-19: an observational study (TEDHICOV). *medRxiv*2021; 2021.03.24.21252635.

[43] DeJong C, Spinelli MA, Okochi H, Gandhi M. Tenofovir-based PrEP for COVID-19: an untapped opportunity?*AIDS*2021; published online March 11. DOI:10.1097/QAD.0000000000002877. 10.1097/QAD.0000000000002877 PMC8243808 33710026

[44] Hernandez-Diaz S, Bateman BT, Straub L, et al. Safety of Tenofovir Disoproxil Fumarate (TDF) for pregnant women facing the COVID-19 pandemic. *Am J Epidemiol*2021; published online April 13. DOI:10.1093/aje/kwab109. 10.1093/aje/kwab109 PMC8083317 33847737

