# Supplementary material for: Effect of Tenofovir Disoproxil Fumarate and Emtricitabine on nasopharyngeal SARS-CoV-2 viral load burden amongst outpatients with COVID-19: A pilot, randomized, open-label phase 2 trial
Source: eClinicalMedicine. 2021 Jun 27;38:100993. doi: 10.1016/j.eclinm.2021.100993 (PMC8235994; doi:10.1016/j.eclinm.2021.100993)
Supplement: Supplementary file 1 [file mmc1.docx]

Supplemental Appendix

Tenofovir disoproxil fumarate and emtricitabine to attenuate R_0_ in outpatients with recent mild-to-moderate COVID-19: a pilot randomised, open label, controlled, phase IIb trial

Methods

Reverse Transcriptase Polymerase Chain Reaction methods

All nasopharyngeal swab samples collected at private diagnostic laboratories around the Caen area were centralised at the Department of virology, Caen University hospital. The same Real-time PCR assay was used for the three samples throughout the study: Allplex^TM^ 2019-nCoV Assay COVID-19 detects SARS-CoV-2 by amplification of RdRp gene, E gene, and N gene according to WHO's recommended protocol. Because the nucleo(t)sides analogues combination targets the RdRp enzyme, we planned to use SARS-CoV-2 RdRp gene Ct. All nasopharyngeal swab samples collected at the Orléans area were centralised at the Department of virology, Orléans Regional hospital. The same Real-time PCR assay was used for the three samples throughout the study: TaqPath™ COVID-19 Multiplex RT-PCR Thermofisher, which detects SARS-CoV-2 by amplification of ORF1ab gene, S gene, and N gene. Because the S gene may be negative for some new SARS-CoV-2 variants, we planned to use SARS-CoV-2 N gene Ct.

In addition, we conducted a post-hoc sensitivity analysis in which all samples from Orléans were centralised at the Department of virology, Caen University hospital using SARS-CoV-2 RdRp gene Ct. When duplicate sample was not available or could not be amplified, we used the amplification of N gene for the three samples (baseline, day 4 and day 7) throughout the study. Positivity was recorded when an amplification curve with a Cycle threshold (Ct) < 40 was detected.

Results

The Supplemental Figure 1 displays the virologic outcomes at day 4 and day 7 of the *post hoc* centralised nasopharyngeal samples RT-PCR for the RdRp gene of SARS-CoV-2. Samples were lost or could not be amplified in 7/30 cases in the standard of care group and 9/30 in the treatment group. The RT-PCR for the N gene performed on fresh samples were used instead.

Amongst patients who received tenofovir disoproxil fumarate, the difference from standard of care in the increase in Ct RT-PCR from baseline was 2.0 (95% confidence interval [-0.7 to 4.7], p=0.15) at day 4 and 3.0 (95% CI [0.6 to 5.4], p=0.014) at day 7.

Supplemental Figure 1. Variation of Ct RT-PCR for SARS-CoV-2 in centralised nasopharyngeal samples by study visit according to allocated groups
